# A Case of First Branchial Cleft Fistula Presenting with an External Opening on the Root of the Helical Crus

**DOI:** 10.1155/2018/4215802

**Published:** 2018-01-30

**Authors:** Sung Il Cho

**Affiliations:** Department of Otolaryngology-Head and Neck Surgery, Chosun University School of Medicine, Gwangju, Republic of Korea

## Abstract

**Background:**

First branchial cleft anomalies (FBCA) are rare clinical entities of the head and neck. Typically, the tract of the FBCA begins in the external auditory canal and ends in the postauricular or submandibular region.

**Case Presentation:**

We present a case of a 23-year-old man who had a first branchial cleft fistula with atypical opening on the root of the helical crus. Complete excision of the tract, including the cuff of surrounding cartilage, was performed. Histopathology revealed a fistular tract lined with squamous epithelium. To our knowledge, this is the first case to be reported of type I FBCA with an opening on the root of the helical crus. The low incidence and varied presentation often result in misdiagnosis and inappropriate treatment.

**Conclusions:**

In the patients with FBCA, careful recognition of atypical variants is essential for complete excision.

## 1. Background

First branchial cleft anomalies (FBCA) are very rare and account for less than 10% of all branchial cleft anomalies. Work reported a classification system based on embryology and histology [1]. Type I anomalies are ectodermal in origin and arise from the duplication of the membranous external auditory canal. Type II anomalies are ectodermal and mesodermal in origin and arise from the duplication of the membranous and cartilaginous external auditory canal [2]. The superior openings of these anomalies are commonly located on the floor of the external auditory canal. Herein, we report the first case of a branchial cleft fistula with the superior opening located on the root of the helical crus, extending into the postauricular crease.

## 2. Case Presentation

**Figure 1:**
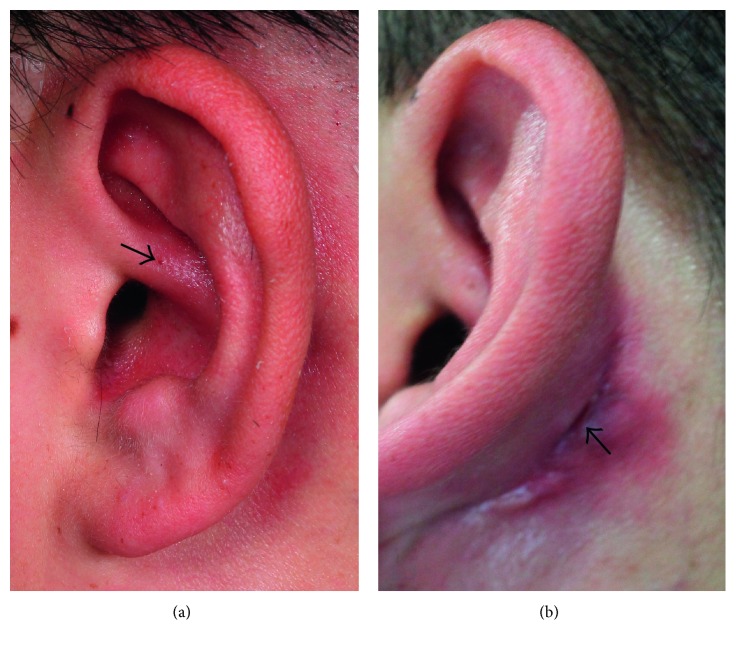
The opening of the fistula tract is seen on the root of the helical crus (a). A lower opening is visible in the postauricular crease (b).

**Figure 2:**
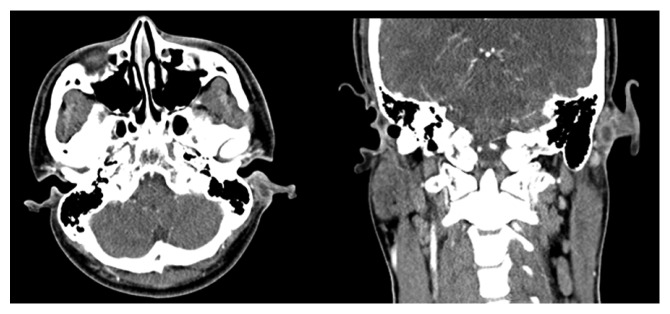
A CT scan with contrast shows a left rim-enhancing cyst in the postauricular crease.

**Figure 3:**
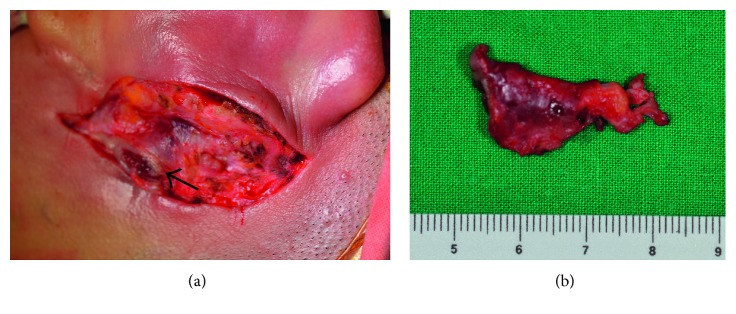
Surgical findings of the fistula tract. The tract and the opening in the postauricular crease (arrow) were excised (a). The fistula tract after total removal (b).

**Figure 4:**
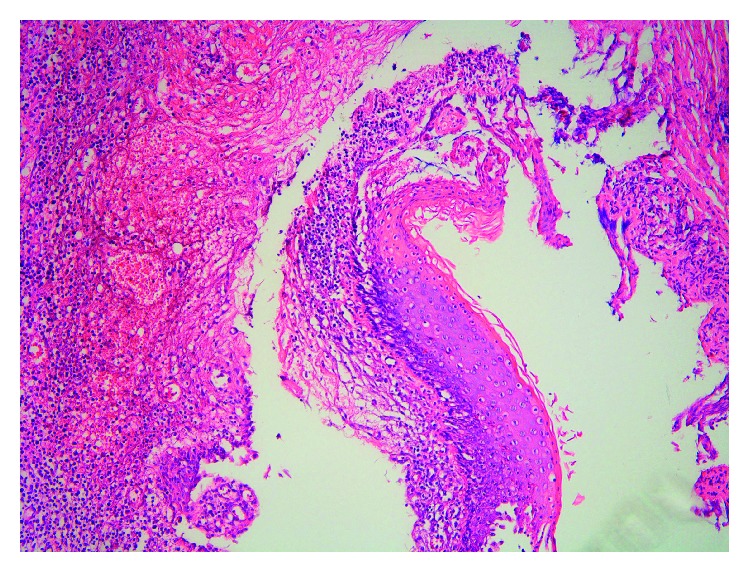
The fistula was lined with squamous epithelium, with features suggestive of a branchial cleft fistula (×100, hematoxylin and eosin stain).

A 23-year-old man was referred to our clinic with postauricular drainage and swelling. He had a 10-year history of recurrent swelling in the postauricular area. Physical examination revealed a fistulous tract extending from the root of the helical crus to the postauricular crease. The lower opening in the postauricular area was surrounded by a reddish cystic lesion (Figure 1). The patient had no history of facial weakness. A preoperative computed tomography (CT) scan showed a left rim-enhancing cyst in the postauricular crease (Figure 2). Postauricular discharge was purulent and was treated with antibiotics. On suspicion of a branchial cleft fistula, surgical resection of the fistula was scheduled. A postauricular incision including an ellipse around the lower opening was performed. The tract and lower opening, including the surrounding skin, were excised. The tract was traced anteriorly to the helical crus and a portion of helical cartilage was excised in continuity with the tract (Figure 3). The tract was identified as superficial to the facial nerve. Histopathology revealed that the excised tissue was a fistula lined with squamous epithelium (Figure 4). The patient received intravenous antibiotics for 3 days and oral antibiotics for 2 weeks as a precautionary measure. The wound completely resolved after 1 month. During the 24-month follow-up, no sign of recurrence was found.

## 3. Discussion

FBCAs occur due to abnormal development of the branchial apparatus during weeks 4 to 7 of fetal life. The structures that develop from the first branchial cleft are the cavum conchae, external auditory canal, and outer layer of the tympanic membrane. Therefore, FBCAs are closely related to these structures [3]. Incomplete closure causes various types of first branchial cleft anomalies such as cysts, sinuses, and fistulas [4]. The embryology of these anomalies reveals that the lesions are derived from the dorsal portion of the first branchial groove and lie between the derivatives of Meckel's and Reichert's cartilages. Therefore, the opening of the tract could occur anywhere, including in the external auditory canal, the postauricular region, or the neck over the angle of the mandible [5].

Type I anomalies are purely ectodermal origin and contain squamous epithelium; this type of anomaly develops medial to the concha, often extending into the postauricular crease and ending in a cul-de-sac at the osseous-cartilaginous junction of the external auditory canal. These anomalies are usually superficial to the branch of the facial nerve. Type II anomalies are ectodermal and mesodermal in origin and thus contain adnexal skin structures and cartilages. The openings of the anomalies are usually below the angle of the mandible and extend toward the inferior part of the external auditory canal; the anomalies pass superficial to, deep to, or between the branches of the facial nerve [6, 7]. Type I FBCAs are very rare, while type II FBCAs are more frequent [8].

In the present case, the histopathological characteristics of the tract extending from the root of the helix to the postauricular crease showed only squamous epithelium and no cartilage. There was no visible opening of the fistula in the external auditory canal. The lesion was superficial to the facial nerve. On the basis of these characteristics, we classified the anomaly as a type I FBCA. The most frequent clinical feature of a type I FBCA is ear discharge or recurrent periauricular swelling. The rare incidence and varied presentation often lead to misdiagnosis and inappropriate treatment, such as multiple incision and drainage procedures. Inappropriate treatments and recurrent infection may result in incomplete excision. When there is a history of prior infection, the recurrence rate of FBCAs can be as high as 14–22% [9]. Definitive treatment for an FBCA is surgical excision of the entire anomaly, including a small amount of surrounding skin or cartilage. Accordingly, we excised the anomaly, including a small portion of the surrounding cartilage.

## 4. Conclusions

Atypical variants of FBCA exist, and various presentations make diagnosis and treatment difficult. Careful examination and dissection are crucial to avoid rest of the anomalies.
